# Clinical evaluation of an in-house panfungal real-time PCR assay for the detection of fungal pathogens

**DOI:** 10.1007/s15010-020-01395-7

**Published:** 2020-02-12

**Authors:** Iris Camp, Gabriele Manhart, Claudia Schabereiter-Gurtner, Kathrin Spettel, Brigitte Selitsch, Birgit Willinger

**Affiliations:** 1 Division of Clinical Microbiology, Department of Laboratory Medicine, Medical University of Vienna, Vienna, Austria; 2 Present Address: Institute for Medical Biochemistry, University of Veterinary Medicine Vienna, Vienna, Austria; 3 Present Address: Ingenetix GmbH, Vienna, Austria

**Keywords:** Broadrange, Real-time PCR, Invasive fungal infections, ITS2, Panfungal

## Abstract

**Purpose:**

Due to an increasing incidence of invasive fungal infections, the availability of reliable diagnostic tools for the fast detection of a wide spectrum of fungal pathogens is of vital importance. In this study, we aimed to conduct an extensive clinical evaluation of a recently published in-house panfungal PCR assay on samples from suspected invasive fungal infections.

**Methods:**

Overall 265 clinical samples from 232 patients with suspected invasive fungal disease (96 deep airway samples, 60 sterile fluids, 50 tissue biopsies, and 59 blood samples) were included. All samples underwent standard culture-based diagnostics and were additionally analyzed with our panfungal PCR assay.

**Results:**

Overall, 55.1% of agreement between culture and the panfungal PCR was observed; in 17% of all samples partial concordance was noted, while results between culture and our PCR assay were not in agreement in 27.9%. Our panfungal assay performed better in samples from normally sterile sites, while samples from the deep airways yielded the highest rate of discordant (39.6%) results. In two tissue and three blood samples an invasive pathogen was only detected by PCR while cultures remained negative.

**Conclusion:**

In combination with routine methods, our panfungal PCR assay is a valuable diagnostic tool. Patients at risk for invasive fungal infections might profit from the reduced time to pathogen identification.

## Introduction

Advances of modern medicine have led to the increased availability and use of immunosuppressant drugs, and an ever growing number of immunosuppressed patients. These patients are at high risk for invasive fungal infections, and infections with rare fungal pathogens are gaining relevance [1–11]. Thus, new diagnostic tools with the ability to detect common but also unusual and emerging fungal pathogens are urgently needed. Since disease progression—especially in immunocompromised patients—can be rapid, early detection is important for a favourable clinical outcome [12, 13]. Currently, culture methods and histology still are considered to be the gold standard for the diagnosis of fungal infections. However, histology often does not allow the discrimination between distinct fungal species, and—due to the slow growth of most fungal pathogens—culture methods can entail a considerable diagnostic delay. Furthermore, routine culture is not possible for some fungal species like *Pneumocystis jirovecii* [14–16], or has a low sensitivity as was reported for *Mucorales* [17, 18]. Thus, in addition to these traditional methods, molecular tests are widely used for the diagnosis of fungal infections today [19–22]. Species- or genus-specific PCR assays only target a narrow spectrum of pathogens, and therefore can only be used if evidence for the infection with a certain pathogen already is present. Panfungal real-time PCR on the other hand enables the unspecific detection and quantification of all fungal DNA present in a clinical sample. For species identification, obtained amplicons then have to be sequenced. To the best of our knowledge, only one panfungal PCR assay currently is CE/IVD certified. The SepsiTest™ is marketed by Molzym (Molzym Molecular Diagnostic, Bremen, Germany), and targets the 16S rRNA gene for bacteria, and the 18S rRNA gene for fungi. In addition to this assay, few research use only (RUO) tests like the Fungiplex Universal RUO PCR kit (Bruker Daltonik, Bremen, Germany) are commercially available, and a number of in-house panfungal assays have been described [21, 23–30]. We recently published a new panfungal HybProbe real-time PCR assay using the LightCycler instrument (Roche), which targets the complete fungal ITS2 region [31]. Due to the broad-range primers and probes, the assay theoretically allows the detection of all fungal pathogens. This approach has the potential to detect microorganisms found less frequently, and to detect uncultivable—or even unknown causative—pathogens of fungal origin. In a validation with 105 clinical samples from 98 patients we already were able to show that our assay is capable of improving the early diagnosis of invasive fungal infections with a sensitivity of 90.4% and a specificity of 79.2% [31]. In the present study we wanted to conduct a larger scale clinical evaluation of our assay as a screening tool for fungal infections.

## Materials and methods

### Clinical evaluation

For the clinical evaluation samples were collected at various departments of the General Hospital of Vienna. Patients were symptomatic high-risk patients (neutropenic patients, patients with leukemia, or patients who underwent thoracic or abdominal surgery) with suspected invasive fungal infections (mainly from the haematology and oncology unit as well as intensive care units). Further inclusion criteria were hospital stay for at least 10 days, broad-spectrum antibiotic therapy, intravascular catheters and/or colonization with *Candida*. A few samples from the ophthalmology department (vitreous fluid and cornea material) were also included in our study. Specimens were taken from normally sterile sites (tissue, sterile fluids, EDTA blood and blood in blood culture bottles) and the deep airways.

Tissues, sterile fluids and samples from the deep airways were divided in two parts; one part was analyzed by routine methods such as microscopy and culture, and the second part was used for DNA isolation and our panfungal PCR assay. To compare our assay’s performance with that of blood culture, a vial of EDTA blood was collected for the panfungal assay additionally to the blood cultures. Species identification was performed using biochemical methods, MALDI-TOF MS, or through the evaluation of macroscopic and microscopic features when dealing with filamentous fungi. If conventional identification was not possible, DNA for sequencing was isolated from cultures. In case of positive panfungal PCR results, amplicons underwent subsequent sequence analysis.

Other tests requested by the treating physicians (e.g. detection of galactomannan, or PCR assays specific for certain species of *Aspergillus* or *Candida*) were performed as part of the diagnostic routine according to the standard operating procedure of our laboratory. All available results were used to aid the interpretation of our data.

### Ethical statement

The study was approved by the ethical committee of the Medical University of Vienna. All patients signed a consent form before samples were collected.

### Culture

To prevent contamination, samples were inoculated in a Laminar flow cabinet class II (BioWizard Silver SL–130 Blue Series, Kojair, Vilppula, Finland). Samples from the deep airways, sterile fluids and tissue samples were inoculated on BBL Sabouraud Dextrose Agar with Chloramphenicol and Gentamicin (SAB, Becton Dickinson, Heidelberg, Germany), ChromAgar Candida Medium (Becton Dickinson, Heidelberg, Germany), Brain–Heart-Infusion-Agar (BHI; Becton Dickinson, Heidelberg, Germany) as well as SAB-broth (Becton Dickinson, Heidelberg, Germany). Deep airway samples were cultured quantitatively (10 µl loop) on SAB agar plates. For sterile fluids and homogenized tissue samples, SAB agar plates were inoculated with Pasteur pipettes (approximately 50 µl). BHI agar and ChromAgar were inoculated with Pasteur pipettes (approximately 50 µl), and 500 µl were used to inoculate SAB-broth. Samples were incubated at 28 °C (BHI agar and SAB-broth) or at 37 °C (SAB agar and ChromAgar) for up to 3 weeks. Whenever possible, samples were examined by KOH and calcofluor white microscopy. Cultured fungal pathogens from samples with negative microscopy results were reported as possible contamination if the growth of a fungus was only noted after unusually long incubation times or if the fungus was only cultured on one medium. The BacT/ALERT 3D system (BioMerieux, Marcy l’Etoile, France) was used for blood cultures. Samples consisting of an aerobic and and anaerobic bottle were incubated for up to 7 days at 36.5–37 °C. From positive blood cultures, a gram stain was performed, and samples were subcultured on solid media.

### DNA extraction

For the mechanical workup of the samples, two kits were used in the first step of the extraction process. For tissues, MagNA Lyser Green Beads (Roche, Basel, Switzerland) were used, while BALs and other fluids were processed with the SeptiFast Lys Kit (Roche). Subsequent DNA extraction from all clinical samples was performed with the High Pure PCR Template Preparation Kit (Roche). DNA was eluted in 100 µl elution buffer. Elution buffer was used as a reagent control.

### Real-time PCR and sequencing

Real-time PCR was performed as described previously [31]. If fungal DNA was detected in a sample by the presence of an amplification curve at 640 nm, the PCR product was sequenced using the ABI PRISM 310 Sequence Detector by BigDye chemistry (Applied Biosystems, Vienna, Austria). Databases used for the identification were the GeneBank database (https://www.ncbi.nlm.nih.gov/blast/), the CBS database (https://www.westerdijkinstitute.nl), and the ITS sequence database by the Centraalbureau voor Schimmelcultures dermatophyte website (https://www.cbs.knaw.nl/dermatophytes/).

## Results

All in all, 265 samples from 232 patients were included in the study. The samples consisted of 96 deep airway samples from 84 patients, 60 samples of various sterile fluids from 59 patients, 50 tissue samples from 43 patients, and 59 EDTA blood samples from 57 patients. From 11 patients, more than one sample type was analyzed. Results of traditional methods and our new assay were in agreement in 146/265 (55.1%) of all samples. In 45/265 (17%) of the samples partial agreement (i.e., agreement on genus level or identification of one of multiple pathogens detected by culture) was observed, while 74/265 (27.9%) of the samples gave discordant results.

### Blood samples

In total, 59 blood samples were investigated using conventional blood culture and our PCR assay (Table 1). Forty-three samples (72.9%) showed concordant results, while results from 16 samples (27.1%) were not in agreement.

**Table 1:** Details of 59 blood samples

	*n*	Blood culture	Panfungal PCR
Concordant results (n = 43)	31	Negative	Negative
	12	*Candida*	*Candida*
		*C. albicans* (*n* = 8)	*C. albicans* (*n* = 8)
		*C. parapsilosis* (*n* = 3)	*C. parapsilosis*(*n* = 3)
		*C. glabrata* (*n* = 1)	*C. glabrata* (*n* = 1)
Discordant results (*n* = 16)	13	Negative	True positive (*n* = 3)
			*C. albicans* (*n* = 1)
			*A. flavus* (*n* = 1)
			*A. fumigatus* (*n* = 1)
			Contaminants (*n* = 10)
			*Y. lipolytica* (*n* = 4)
			*S. cerevisiae* (*n* = 2)
			*C. parapsilosis* (*n* = 1)
			*C. neoformans* (n = 1)
			Uncultured *Malassezia* clone(*n* = 1)
			*Lecanicillium sp*. (*n* = 1)
	1	*C. albicans*	Negative (*n* = 3)
	1	*C. parapsilosis*	
	1	Gram stain: yeast cells / no growth upon subculture	

Test results for blood samples

*n* number of samples

The same pathogen was detected by blood culture and our PCR assay in 12 cases. In 31 samples blood cultures as well as our panfungal PCR assay were negative, even though *C. albicans* was detected in two, and *A. fumigatus* in five cases by the SeptiFast^®^ (Roche)—a commercially available multiplex PCR. In additional five of those negative samples, blood cultures taken 1–5 days before study sampling were positive for *C. albicans* (4x) or *C. neoformans* (1x). In another one of these negative samples, *C. albicans* and *C. glabrata* were detected by a *Candida* specific PCR, which was developed earlier at our institution [32]. For this sample, an amplicon was obtained with our panfungal PCR assay, but sequencing did not produce a valid result.

In 13 samples, the PCR detected fungi which were not detected by culture. Even though some of these results might be due to contamination, PCR results from three samples were confirmed by clinical signs as well as results obtained by additional PCR assays such as the SeptiFast^®^ and our formerly developed *Aspergillus*-specific real-time PCR [32]. SeptiFast^®^ confirmed the presence of *C. albicans* in one, and of *A. fumigatus* in another sample. Furthermore, SeptiFast^®^ and our *Aspergillus*-specific PCR assay detected both *A. flavus* and *A. fumigatus* in the sample positive for only *A. flavus* in our panfungal assay.

Three samples were only positive in blood culture while the PCR remained negative. In two cases *C. albicans* or *C. parapsilosis* grew in blood cultures. In these cases, the blood cultures were taken 2 days ahead of the EDTA blood sample, which might be the reason for the negative PCR result. The Gram stain of the third sample revealed the presence of yeast cells, but the pathogen could not be isolated as all subcultures remained negative. Even the more sensitive *Candida*-specific real-time-PCR-assay did not yield a positive result for this sample.

### Sterile fluid samples

In our study, 60 sterile fluid samples (Table 2) such as cerebrospinal fluid, bile, ascites, vitreous fluids, and others were analyzed. Thirty-six samples (60%) gave concordant results; results from 13 samples (21.7%) were in partial agreement, while no agreement was observed in results obtained from 11 samples (18.3%).

**Table 2:** Details of 60 sterile fluid samples

	*n*	Culture	Panfungal PCR
Concordant results (*n* = 36)	1	Negative	Negative
	26	*Candida*	*Candida*
		*C. glabrata* (*n* = 9)	*C. glabrata* (*n* = 9)
		*C. albicans* (*n* = 8)	*C. albicans* (*n* = 8)
		*C. parapsilosis* (*n* = 4)	*C. parapsilosis*(*n* = 4)
		*C. tropicalis* (*n* = 2)	*C. tropicalis* (*n* = 2)
		*C. dubliniensis* (*n* = 1)	*C. dubliniensis* (*n* = 1)
		*C. krusei* (*n* = 1)	*C. krusei* (*n* = 1)
		*C. lusitaniae* (*n* = 1)	*C. lusitaniae* (*n* = 1)
	4	*A. fumigatus*	*A. fumigatus*
	2	*E. dermatitidis*	*E. dermatitidis*
	1	*F. oxysporum*	*F. oxysporum*
	1	*C. neoformans*	*C. neoformans*
	1	*S. cerevisiae*	*S. cerevisiae*
Partially concordant results (*n* = 13)	10	Mixed culture	
		*C. albicans* + *C. glabrata* (*n* = 3)	*C. glabrata* (*n* = 3; 1 sample:*)
		*C. albicans* + *C. glabrata* (*n* = 1)	*C. albicans* (*n* = 1)
		*C. albicans* + *S. cerevisiae* (*n* = 1)	*C. albicans* (*n* = 1)
		*C. glabrata* + *C. tropicalis* (*n* = 1)	*C. glabrata* (*n* = 1)
		*C. glabrata* + *C. tropicalis* (*n* = 1)	*C. tropicalis* (*n* = 1)
		*C. glabrata* + *C krusei* (*n* = 1)	*C. glabrata* (*n* = 1)
		*C. inconspicua* + *C. lipolytica* (*n* = 1)	C. inconspicua* (*n* = 1)
		*C. parapsilosis* + *Zygosaccharomyces sp.* (*n* = 1)	*C. parapsilosis* (*n* = 1)
	3	*Candida*	*Candida*
		*C. glabrata* (*n* = 1)	*C. dubliniensis* (*n* = 1)
		*C. glabrata* + *C. tropicalis* (*n* = 1)	*C. albicans* (*n* = 1)
		*C. dubliniensis* (*n* = 1)	*C. albicans* (*n* = 1)
Discordant results (*n* = 11)	6	*Candida*	Negative/no valid sequencing result (*n* = 7)
		*C. glabrata* (*n* = 4)	
		*C. krusei* (*n* = 1)	
		*C. parapsilosis* (*n* = 1)	
	1	*A. fumigatus*	
	1	R. arrhizus**	*S. cerevisiae*
	1	*S. cerevisiae*	*C. tropicalis*
	1	*C. glabrata*	*S. cerevisiae*
	1	*C. krusei* + *Candida sp.*	*K. telluris*

Test results for sterile fluid samples

*n* number of samples

*PCR yielded a double sequence

**Culture result was reported as possible contamination

In 35 samples, only one pathogen was detected, and both methods identified the same species. Furthermore, culture and molecular methods gave a negative result for one sample.

Ten of the samples with partial agreement showed growth of two fungi in culture, but our panfungal PCR only managed to detect one of the species. However, double bands—indicating the presence of multiple pathogens—were observed in two of these samples. In three samples, concordance between culture and the PCR assay was achieved on genus level only.

Of the 11 discordant samples, seven yielded positive culture results but remained negative (3x) or did not produce a valid sequencing result (4x) in our panfungal PCR assay. Since KOH and calcofluor white microscopy was negative in all of those samples, and clinical signs and results of additional exams refuted a fungal infection, contamination of the culture is likely. In four samples—all of them being KOH negative—cultures and the PCR assay detected different fungi.

### Tissue samples

Fifty tissue samples were investigated in the course of our study (Table 3). Approximately one third of the tissue samples were obtained from lungs, but also other types of tissue—such as tissue of the ear lobe, sinuses, jaw, and cornea—were included. Thirty samples (60%), including eight negative samples, showed agreement between culture and PCR. Eleven samples (22%) gave partially concordant results; while 9 samples (18%) gave discordant results.

**Table 3:** Details of 50 tissue samples

	*n*	Culture	Panfungal PCR
Concordant results (*n* = 30)	8	Negative	Negative
	11	*Candida*	*Candida*
		*C. albicans* (*n* = 9)	*C. albicans* (*n* = 9)
		*C. parapsilosis* (*n* = 1)	*C. parapsilosis*(*n* = 1)
		*C. dubliniensis* (*n* = 1)	*C. dubliniensis* (*n* = 1)
	5	*A. fumigatus*	*A. fumigatus*
	2	*C. neoformans*	*C. neoformans*
	1	*R. microsporus*	*R. microsporus*
	1	*E. dermatitidis*	*E. dermatitidis*
	1	*B. australiensis*	*B. australiensis*
	1	*S. cerevisiae*	*S. cerevisiae*
Partially concordant results (*n* = 11)	10	Mixed culture	
		*R. microsporus* + *C. parapsilosis* + *C. albicans* (*n* = 1)	*C. parapsilosis* (*n* = 1)
		*C. albicans* + *C. glabrata* (*n* = 3)	*C. albicans* (*n* = 3; 1 sample:*)
		*C. albicans* + *S. cerevisiae* (*n* = 1)	*C. albicans* (*n* = 1)
		*C. glabrata* + *C. albicans*(*n* = 1)	C. glabrata* (*n* = 1)
		*C. dubliniensis* + *C. albicans* (*n* = 1)	*C. dubliniensis* (*n* = 1)
		*S. cerevisae* + *C. krusei* (*n* = 1)	*S. cerevisiae* (*n* = 1)
		*S. cerevisiae* + *C. parapsilosis* (*n* = 1)	S. cerevisiae* (*n* = 1)
		*E. dermatitidis* + *P. lilacinus* (*n* = 1)	*E. dermatitidis* (*n* = 1)
	1	*C. albicans*	*C. lusitaniae*
Discordant results (*n* = 9)	2	*Negative*	*A. fumigatus*
	2	A. fumigatus**	Negative/no valid sequencing result (*n* = 6)
	1	*F. solani*	
	1	*R. mucilaginosa*	
	1	*A. corymbifera*	
	1	*C. glabrata*	
	1	*C. dubliniensis*	*C. cladosporioides*

Test results for tissue samples

*n* number of samples

*PCR yielded a double sequence

**Culture result was reported as possible contamination

In ten of the samples with partial agreement more than one species was detected by culture, but PCR only detected one species. A double sequence—again an indicator for the presence of multiple fungi—was noted in three of these samples. One sample showed concordance on genus level; *C. albicans* was detected by culture, but sequencing indicated *C. lusitaniae*.

In two samples, *A. fumigatus* could only be detected by PCR, while culture remained negative during the entire incubation period. KOH microscopy was positive for both samples, and the PCR results were confirmed using our *Aspergillus* specific real-time PCR. In six samples, fungi grew in culture but the PCR remained negative. Two of these culture results were reported as possible contamination; KOH microscopy was negative for five of six samples, and no KOH microscopy result was available for the sample with growth of *Fusarium solani* species complex; however, it took 4 weeks for the pathogen to grow. This is unusually long and thus might indicate a mere contamination of the culture plate. For one sample, our PCR assay and the conventional methods indicated the presence of different fungi.

### Deep airway samples

Our PCR assay also was evaluated with 96 deep airway samples (Table 4). These consisted of 90 BALs and six tracheal/bronchial secretions. In 37 samples (38.5%), concordant species level identification was achieved with culture and PCR. In 21 samples (21.9%) partial concordance was observed, while results did not match in 38 samples (39.6%).

**Table 4:** Details of 96 deep airway samples

	*n*	Culture	Panfungal PCR
Concordant results (*n* = 37)	21	*Candida*	*Candida*
		*C. albicans* (*n* = 14)	*C. albicans* (*n* = 14)
		*C. dubliniensis* (*n* = 2)	*C. dubliniensis* (*n* = 2)
		*C. kefyr* (*n* = 2)	*C. kefyr* (*n* = 2)
		*C. tropicalis* (*n* = 2)	*C. tropicalis* (*n* = 2)
		*C. lusitaniae* (*n* = 1)	*C. lusitaniae* (*n* = 1)
	13	*Aspergillus*	*Aspergillus*
		*A. fumigatus* (*n* = 12)	*A. fumigatus* (*n* = 12)
		*A. niger* (*n* = 1)	*A. niger* (*n* = 1)
	1	*S. cerevisiae*	*S. cerevisiae*
	1	*G. capitatum*	*G. capitatum*
	1	*S. apiospermum*	*S. apiospermum*
Partially concordant results (*n* = 21)	18	Mixed culture	
		*C. neoformans* + *A. fumigatus* (*n* = 1)	*C. neoformans* (*n* = 1)
		*A. fumigatus* + *C. glabrata* (*n* = 1)	*A. fumigatus* (*n* = 1)
		*A. fumigatus* + *C. glabrata* + *C. krusei* (*n* = 1)	*A. fumigatus* (*n* = 1)
		*A. fumigatus* + *S. cerevisiae* (*n* = 1)	*A. fumigatus* (*n* = 1)
		*Aspergillus sp.* + *C. albicans* (*n* = 1)	*A. fumigatus* (*n* = 1)
		*A. fumigatus* + *C. albicans* + *C. glabrata* (*n* = 1)	*C. albicans* (*n* = 1)
		*A. fumigatus* + *C. glabrata* (*n* = 1)	*C. glabrata* (*n* = 1)
		A. fumigatus* + *C. albicans* (*n* = 2)	*C. albicans* (*n* = 2)
		A. fumigatus* + *C. tropicalis* + *C.glabrata* (*n* = 1)	*C. tropicalis* (*n* = 1)
		*A. nidulans* + *S. cerevisiae* + *C. albicans* (*n* = 1)	*S. cerevisiae* (*n* = 1)
		*A. niger* + *C. tropicalis* (*n* = 1)	*C. tropicalis* (*n* = 1)
		*C. glabrata* + *C. albicans* (*n* = 3)	*C. glabrata* (*n* = 3)
		*C. dubliniensis* + *S. cerevisiae* (*n* = 1)	*C. dubliniensis* (*n* = 1)
		*C. albicans* + *C. glabrata* (*n* = 1)	*C. albicans* (*n* = 1)
		*C. albicans* + *G. candidum* (*n* = 1)	*C. albicans* (*n* = 1)
	3	*Aspergillus*	*Aspergillus*
		*A. candidus* (*n* = 1)	*Aspergillus sp.* (*n* = 1)
		*A. candidus* (*n* = 1)	*A. persii, A. sclerotiorum, A. bridgeri* (*n* = 1)
		*A. terreus* (*n* = 1)	*A. fumigatus* (*n* = 1)
Discordant results (*n* = 38)	4	Negative	*C. glabrata* (*n* = 2)
			*M. restricta* (*n* = 1)
			Uncultured Lemonniera clone (*n* = 1)
	10	*A. fumigatus* (*n* = 5)	Negative (*n* = 10)
		A. niger* + C. albicans* (*n* = 1)	
		*C. neoformans* (*n* = 1)	
		*C. lipolytica* (*n* = 1)	
		*C. albicans* + *G. capitatum* (*n* = 1)	
		Hyalohyphomycet (*n* = 1)	
	14	*Aspergillus*	
		*A. fumigatus* (*n* = 4)	*C. albicans* (*n* = 1)
			*C. lusitaniae* (*n* = 1)
			*S. commune* (*n* = 1)
			*P. spinulosum, P. thomii, P. purpurascens, P. glabrum* (*n* = 1)
		A. fumigatus* (*n* = 2)	*M. restricta* (*n* = 1)
			*C. cladosporioides* (*n* = 1)
		*A. fumigatus* + *A. niger* (*n* = 1)	Penicillium sp.** (*n* = 1)
		*A. fumigatus* + C. krusei (*n* = 1)	*C. albicans* (*n* = 1)
		A. fumigatus* + Penicillium sp.* (*n* = 1)	Cladosporium sp.** (*n* = 1)
		A. fumigatus* + C. dubliniensis* (*n* = 1)	A. terreus** (*n* = 1)
		*A. terreus* (*n* = 3)	*C. dubliniensis* (*n* = 1)
			C. cladosporioides** (*n* = 1)
			*C. cladosporioides, uncultured Davidiella clone* (*n* = 1)
		A. niger* (*n* = 1)	*P. chrysogenum* (*n* = 1)
	6	*Candida*	
		*C. albicans* (*n* = 4)	*M. restricta* (*n* = 2)
			Cladosporium sp.** (*n* = 2)
		*C. glabrata* (*n* = 1)	*C. krusei* (*n* = 1)
		*C. norvegensis* + *C. parapsilosis* (*n* = 1)	C. krusei** (*n* = 1)
	1	F. oxysporum*	*P. thomii, P. spinulosum, P. glabrum*
	1	Penicillium sp.*	*C. albicans*
	1	*L. corymbifera*	*C. albicans*
	1	*G. capitatum*	*C. albicans*

Test results for deep airway samples

*n* number of samples

*Culture result was reported as possible contamination

**PCR yielded a double sequence

Most of the concordant samples contained various yeasts. In 13 samples *Aspergillus sp.* were detected by culture and PCR, and *S. apiospermum* was found in one sample by both methods.

In 18 of the partially concordant samples, culture yielded more than one fungal species, but our panfungal PCR only was able to detect one of the species. For example, *A. fumigatus* and *C. neoformans* grew on the culture plate, but only *C. neoformans* was detected by our PCR assay. Since the patient did not show any clinical signs of aspergillosis, *A. fumigatus* was probably a contaminant. In three samples—all with growth of *Aspergillus sp*., agreement was at the genus level only.

Of the discordant samples, four did not show any growth in culture even though the presence of fungi was detected by PCR, and PCR remained negative in 10 samples with positive culture results. The presence of different fungi was detected by culture and PCR in 24 samples. In six of these 24 samples, sequencing of the PCR amplicon yielded a double sequence.

In four of the discordant samples, the skin colonizer *Malassezia restricta* was detected by PCR. In two of these four patients, cultures were positive for *C. albicans*, while *A. fumigatus* grew in one sample, and the culture remained negative for the fourth patient. No clinical signs of fungal infection were detected for any of the four patients, even though KOH microscopy gave a positive result for one of the patients with *C. albicans*. Thus, contamination of the sample might be possible. In six cases, PCR detected airborne contamination of the samples due to *Cladosporium spp*. None of these samples belonged to patients with invasive mycosis, thus results were interpreted as contamination/colonization, even though cultures showed growth of *A. fumigatus* (2x), *A. terreus* (2x), or *C. albicans* (2x).

## Discussion

As traditional methods for the diagnosis of invasive fungal diseases are often time consuming, the significantly reduced time to result achieved with molecular methods poses a large benefit, especially in emergency settings when fast results are necessary. Other benefits of molecular diagnostic tests are that they are entirely objective and that—in contrast to histology or KOH microscopy—less diagnostic experience is required. Furthermore, compared to culture, notably less specimen material is needed for correct identification. A number of specific PCR assays designed for the detection of one or multiple fungal pathogens are available today [33, 34]. However, for the detection of pathogens which are not included in the panel of specific assays, or in cases in which clinical evidence does not help to narrow down the number of possible pathogens, a panfungal PCR assay can be instrumental for the correct diagnosis.

In our study, the real-time PCRs were performed in parallel to conventional diagnostics for sterile fluid samples, tissue samples and deep airway samples. EDTA blood samples were analyzed on the day they arrived in our laboratory. Even though we requested the EDTA blood and the blood culture sample to be taken from the same blood draw, clinicians did not always comply with this request. Unfortunately, in 25 of the 59 blood samples, sampling dates differed. This was the case for 19 samples that gave concordant culture and PCR results (12 negative samples, six of the samples positive for *C. albicans*, and one sample positive for *C. parapsilosis*). Additionally, four of the samples in which the PCR assay detected only contaminants while cultures remained negative were not obtained on the same day; this was also the case for the two culture-positive but PCR-negative samples. We decided to include the 19 concordant samples as well as the four samples in which only contaminants were detected by PCR since the results are comprehensible despite the difference in sampling dates. Furthermore we did not want to withhold the two cases with positive blood cultures (for either *C. albicans* or *C. parapsilosis*) but negative PCR, as they show the limits of our assay’s sensitivity, which is in line with the parameters reported for other in-house panfungal assays. These tests show sensitivities ranging from 69 to 96% [21, 23, 26–28, 30], and consist of up to seven individual multiplex PCRs[28]. The sensitivity of a panfungal PCR assay generally is lower than that of an assay targeting specific pathogens.

Nevertheless, we observed a high number of sequencing results that were in concordance with the results obtained by traditional methods. The diagnostic potential of our assay is especially highlighted by the cases with clinical evidence for an invasive fungal infection in which the underlying pathogen was identified with our panfungal assay while cultures remained negative. For instance, we observed two cases of fungus balls with *A. fumigatus* in the maxillary sinuses (Table 3). Furthermore, our assay was able to detect fungal pathogens in three blood samples even though the blood cultures taken from these patients remained negative (Table 1). Two of these three cases were patients with invasive infections caused by *Aspergillus spp*. Since molds often are not detected by blood culture systems, the use of a molecular or biomarker-based test should always be considered in patients when an invasive infection with filamentous fungi, e.g., an invasive aspergillosis is suspected. Steinmann et al. for example showed that the SeptiFast^®^ is a valuable diagnostic test for the detection of *A. fumigatus *[35]. Also for the detection of candidemia molecular tests are important add-ons to culture-based diagnostics, since the sensitivity of blood culture for *Candida* spp. is low [36], and the time to positivity and subsequent species identification often exceeds 48 h. A limitation of a panfungal molecular assay is that no information on possible resistance to antifungals is obtained. Thus, culture should always be performed.

In our study, *Fusarium sp.* was detected by culture in two samples from patients with corneal ulcerations (Tables 2, 3). In one case, the panfungal PCR assay remained negative (Table 3), but since it took unusually long for the pathogen to grow in culture, it is possible that the positive culture was the result of a contaminated culture plate. In the second case, both culture and our PCR assay detected *Fusarium oxysporum* species complex (Table 2). In this case, the panfungal assay reduced the time to detection from 1 week to 2 days. Since delayed therapy of eye fusariosis can have dramatic consequences—e.g., the necessitiy of enucleation—the assay’s ability of rapid detection can be crucial for preserving tissue and quality of life.

Both culture and PCR detected *Cryptococcus neoformans* in a sample of cerebrospinal fluid (Table 2) and two tissue samples (Table 3). The lethality rate of invasive cryptococcosis is estimated to be at least 63% [37]. Therefore, a short time to result has major implications on the management of the patient and the therapeutic outcome.

Mucorales are another group of emerging fungal pathogens that require fast diagnosis. Histology results indicated the presence of *Mucorales* in one of the patients included in our study. Specimens were taken from the gluteal fat/muscle at two different time points (Table 3). While growth of *Rhizopus microsporus* was observed in both culture samples, PCR only detected the pathogen in one sample. *C. albicans* and *C. parapsilosis* were present in cultures from the second sample additionally to *R. microsporus.* In this sample, PCR was only able to detect *C. parapsilosis*. If more than one species is present in a sample, broad-range PCR assays generally encounter a problem since the presence of multiple amplicons leads to overlapping sequences which can hamper pathogen identification. Therefore it is not surprising that in samples containing multiple fungal pathogens, our assay only was able to detect one of the pathogens.

The interpretation of the results obtained by a panfungal assay can be challenging, as sample type and the sampling location have to be taken into account. *Malassezia spp.* for example are opportunistic skin colonizers and *Candida spp.* are frequently found as transient colonizers in the human respiratory tract. Thus, these fungi might be detected in samples without any clinical significance. Molds like *Cladosporium sp.* and *Penicillium sp.* are environmental contaminants, and their presence in airway samples of immunocompetent patients usually does not reflect an invasive fungal infection. As reflected by results of our study, a panfungal PCR assay might be of limited use in non-sterile samples from the deep airways. However, in selected cases such an assay might provide meaningful results. For example, *S. apiospermum* was detected by culture and our PCR assay in one of the BAL samples, and *C. neoformans* was detected by both methods in another sample (Table 4). In both cases PCR results were available before growth was noted on culture plates.

Due to its high sensitivity and universal character the panfungal assay is prone to contamination. Since certified fungal-DNA-free plastic ware and ultra pure reagents are not commercially available it can be difficult to distinguish between low copy numbers of pathogen DNA and a mere contamination. Thus, results should always be interpreted with care and in combination with other parameters. It is of crucial importance to review culture and PCR results in conjunction with histologic findings and clinical data to confirm the presence of a fungal infection.

Microscopy is a valuable tool to detect the presence of fungi in clinical samples and thus can help interpreting discordant results. For example, a BAL sample taken from a patient with hemoptysis was positive for *A. fumigatus* in culture, but the mould was not detected by our PCR assay (Table 4). Since the presence of fungi was confirmed by KOH microscopy, contamination of the culture with *A. fumigatus* could be ruled out. On the other hand, a negative microscopy result could reinforce the conclusion that a PCR-positive but culture-negative sample is the result of a contamination.

## Conclusion

Our results show that the diagnostic value of panfungal PCR assays might be hampered by the presence of fungal airway colonizers when samples from the deep airways are examined. However, a panfungal assay is useful for the detection of invasive fungal pathogens in samples from sterile sites, especially when rare fungal pathogens are suspected, or when a wide spectrum of infectious agents should be covered. Since the sensitivity of a broad spectrum PCR assay can not match that of a specific PCR assay, a specific assay should be carried out whenever pathogens are suspected for which such an assay is available. A combination of panfungal and specific assays can be very useful in cases without suspicion for a specific fungal pathogen. A negative result obtained with a broad spectrum assay can never rule out a fungal infection. However, in positive cases, a panfungal PCR can accelerate the diagnosis of an invasive fungal infection in a considerable manner. Since invasive fungal infections are marked by high lethality rates, such a PCR assay is a valuable and maybe even crucial diagnostic tool in a world that experiences a rise of both incidence and variety of fungal infections.
